# Abortion after diagnosis of fetal anomaly: Psychometric properties of a German version of the individual level abortion stigma scale

**DOI:** 10.1371/journal.pone.0197986

**Published:** 2018-06-12

**Authors:** Franz Hanschmidt, Michaela Nagl, Johanna Klingner, Holger Stepan, Anette Kersting

**Affiliations:** 1 Department of Psychosomatic Medicine, University of Leipzig, Leipzig, Germany; 2 Department of Obstetrics, University of Leipzig, Leipzig, Germany; University of California, San Francisco, UNITED STATES

## Abstract

**Background:**

Diagnosis of fetal anomaly is a significant life event and social stigma can negatively impact on the well-being of women opting for an abortion. This study investigated the psychometric properties of a measure of stigma among women who had had an abortion after diagnosis of fetal anomaly in a German setting.

**Methods:**

The Individual Level Abortion Stigma (ILAS) scale was translated into German. Psychometric properties of the ILAS scale were examined among 130 women with a history of an abortion after diagnosis of fetal anomaly. Individual and situational factors associated with stigma in the context of an abortion after diagnosis of fetal anomaly were explored.

**Results:**

Factor analysis suggested a four-dimensional structure of the German version of the Individual Level Abortion Stigma scale (Cronbach’s α, .83–.91), corresponding to the subscales of the original scale. Test-retest reliability was acceptable for the worries about judgment subscale, the self-judgment subscale, and the community condemnation subscale, but less convincing for the isolation subscale. Associations between the subscales and measures of depression, self-esteem and secrecy were found in directions consistent with theory. Women who did not perceive their fetus to have a low survival chance and women whose fetus was at higher gestational age reported higher levels of stigma, whereas higher perceived partner support was associated with lower levels of stigma.

**Limitation:**

Generalizability of study results was limited, as participants were recruited from one clinic in Germany and the study had a response rate of 46.5%.

**Conclusions:**

The ILAS subscales are largely reliable and valid measures to assess stigma among women who have had an abortion after diagnosis of fetal anomaly. Suggestions for improving the assessment of stigma experienced in this population are outlined. The scales can be useful in research aiming at investigating psychological outcomes of abortion after diagnosis of fetal anomaly and improving care structures.

## Introduction

Recent advances in prenatal diagnostics allow for an increasing number of fetal anomalies to be diagnosed [1], and a number of women who receive a diagnosis of fetal anomaly decide to terminate their pregnancy [2–5]. In Germany, it is estimated that the prevalence of abortions after diagnosis of fetal anomaly was 4.4 (95% CI: 3.5–5.4) per 1000 births in 2014 [6]. For affected women, the diagnosis of a fetal anomaly is often unexpected and experienced as a devastating loss of a normal pregnancy [7,8]. While most women believe that their decision to terminate the pregnancy is right [9], it is frequently marked by ambivalence. For example, unsure diagnosis and prognosis regarding the child’s as well as the parents’ future quality of life can complicate the process of decision-making [7]. Feelings of sadness over the fetal loss may further contribute to ambivalence experienced by women having an abortion after diagnosis of fetal anomaly [7]. The period after the event can be marked by an intensive grief reaction and psychological distress [1,10–13]. However, psychological reactions are comparable to those experienced by women after other prenatal losses and there is no evidence that abortion harms women’s health [14,15].

Feelings of negative self-judgement such as guilt, shame or self-blame are commonly reported by women after fetal loss (e.g., in the case of stillbirth or miscarriage), who may perceive the fetal loss as a personal reproductive failure [16,17]. In the case of abortion after diagnosis of fetal anomaly, the social stigma attached to abortion has been identified as another source of negative judgement, which is the focus of this study [7,18,19]. In many socio-cultural contexts, the legitimacy and morality of abortion are contested, and women who make use of this option may be negatively stereotyped [20]. Under such circumstances, abortion stigma can negatively impact on the well-being of women who opt for an abortion after diagnosis of fetal anomaly [7,18,19].

Stigma describes a social process in which members of disadvantaged social groups experience negative stereotyping, separation, discrimination and status loss [21]. Theoretical conceptualizations propose that abortion stigma can manifest across three different dimensions in affected individuals: perceived, internalized and enacted stigma [22]. Perceived stigma refers to a woman’s perception of devaluing social attitudes related to her abortion and the expectation that these attitudes lead to discriminatory actions. Internalized stigma results when a woman accepts abortion-related devaluing social norms, beliefs, and attitudes and incorporates them into her self-image. This process can manifest as negative feelings such as guilt and shame. Finally, enacted stigma describes actual experiences of discrimination stemming from a woman’s abortion experience [22,23]. Experiences of abortion stigma have been found to vary dependent on contextual and individual factors such as legislation, religion/religiosity, ethnic affiliation or social support [20].

Studies involving women who had had abortions after diagnosis of fetal anomaly, due to an unintended pregnancy or for other reasons have linked stigma to women’s need to keep the abortion a secret, increased psychological distress and somatic symptoms [24–28]. The vast literature on other stigmatized conditions (i.e., obesity or mental illness) suggests additional negative consequences of abortion stigma for women’s well-being, such as impaired self-esteem [29,30].

Despite the significance of stigma for women‘s abortion experience, there are few valid measures to assess abortion stigma in women who have had abortions. Rice et al. [31] reported on the development of several scales for young women assessing attitudes and perceived stigma (excluding internalized stigma), around pregnancy decisions including abortion. Cockrill et al. [26] developed the Individual Level Abortion Stigma (ILAS) scale that measures abortion stigma in women who have had an abortion across four independent dimensions or subscales: worries about judgment, isolation, self-judgment and community condemnation. It was designed to capture multiple dimensions of women’s experiences of stigma not only at the time of the abortion procedure, but also later. The scale showed good psychometric characteristics in terms of internal consistency and factorial validity. Abortion stigma as measured by the ILAS was associated with secretive behaviors, providing further indication of validity.

However, the ILAS scale has not been validated among women who have had an abortion after diagnosis of fetal anomaly. The psychometric properties have so far only been tested in one study involving a heterogeneous sample with regard to reasons for which women sought an abortion [26]. It is important to analytically separate abortions after diagnosis of fetal anomaly from abortions for other reasons (e.g., abortions for socio-economic reasons), as the personal and cultural meaning being attributed to them is likely to differ [14,19,32,33]. This may also alter women’s experiences of stigma. First, abortions after diagnosis of fetal anomaly usually involve wanted pregnancies, implying a different psychological experience of the pregnancy and the abortion [14]. Second, some authors have argued that abortions based on maternal health or fetal indications are more socially accepted than abortion for other reasons such as socio-economic [32,34]. Women receiving the diagnosis of a fetal anomaly may be perceived as having no other choice than to terminate the pregnancy [33]. Indeed, survey data suggest that 89.4% of the German public support legal access to abortion for a fetal anomaly, as opposed to 41.7% supporting legal access to abortion for a pregnant single woman who does not want to raise the child alone [35]. The degree of stigma experienced by women having abortions after diagnosis of fetal anomaly might thus be lower compared to women having abortions for other reasons such as socio-economic.

Given that many abortions after diagnosis of fetal anomaly occur after the first trimester [36], affected women are also more likely to be confronted with a different legal context. Under current German law, abortion on women’s request is limited to 12 weeks post-conception. After this period, women seeking abortion after diagnosis of fetal anomaly need to obtain medical third-party consent (i.e. the continuation of the pregnancy has to pose a serious threat to maternal (mental) health, according to medical opinion) [37].

Against this background, valid measures are warranted to ensure accurate assessment of experiences of stigma in diverging groups of women having abortions for different reasons. The aim of this study was to investigate the psychometric properties of the ILAS scale in a German sample of women who had had an abortion after diagnosis of fetal anomaly. We further sought to explore socioeconomic and abortion-related factors associated with women‘s experience of stigma.

## Methods

All data were collected in a cross-sectional study using self-administered questionnaires and database information taken from the register of the Department of Obstetrics (University of Leipzig). Details of the same study pertaining to the methods and the sample characteristics have been published elsewhere [28,38].

### Recruitment and participants

Potential study candidates were identified by searching the register of the Department of Obstetrics of the University of Leipzig. Women were eligible for inclusion in the study if they had had an abortion following the diagnosis of a fetal anomaly 1 to 7 years before the time of assessment and were at least 18 years old at the time of assessment. All potential study participants were contacted by mail by the head of the department and invited to fill out the appended survey package. The survey package contained information about the study, informed consent sheets, a prepaid return-envelope and the study questionnaire in paper-pencil form. Study participation was voluntary and no compensation was paid. The study was conducted according to the ethical standards of the Declaration of Helsinki and was approved by the Ethical Review Committee of the University of Leipzig.

### Assessment

#### Sociodemographic and abortion-related variables

The questionnaire contained several questions regarding women’s demographic and socioeconomic situation as well as abortion-related questions that were explicitly created for the purpose of this study. Perceived partner support at the time of the abortion was assessed by a single item (“How did you perceive the support from your partner at the time of the TOPFA/during the time after the TOPFA?”; 5-point Likert scale; “no support at all” to “very strong support”). An open-ended question on women’s reason for the abortion was coded to obtain information about whether women’s decision for the abortion was informed by perceived low survival chance of the fetus (e.g. “[..] child had low survival chance.”, “[..] not clear, whether child would survive the necessary surgery.”). Coding was done by the first and the third author (FH and JK) (Cohen’s kappa = 0.93) and disagreements were revisited and decided by the first author. Additionally, patient register data of the Department of Obstetrics (University of Leipzig) was reviewed to obtain obstetric information about participants’ abortion date, gestational age and the type of the fetal anomaly.

#### Individual Level Abortion Stigma (ILAS) scale

Women’s experiences of abortion-related stigma were investigated with the ILAS scale, which had originally been developed by Cockrill et al. [26]. Responses are registered on Likert-scale items with different response categories (for full item wording and response categories see Results section). The ILAS scale consists of 20 items that assess women’s individual level of stigma at the time of their abortion and after across four subscales or dimensions: worries about judgment (7 items), isolation (6 items), self-judgment (5 items) and community condemnation (2 items). Items on the worries about judgment subscale pertain to women’s concern about negative judgment, such as distancing or gossip, from other people in general as well as “loved ones”. The isolation subscale includes items that measure women’s perception and experiences of support from significant others. The self-judgment subscale includes “predominantly negative feelings towards oneself because of the abortion” ([26], p. 83). The community condemnation subscale requests respondents to estimate the number of people in their community (city or town) holding strong negative views of abortion, thus measuring perceptions of stigma at the community level. Scores are obtained by calculating mean scores on the total scale and individually for the subscales. Items are appropriately recoded so that higher scores reflect higher levels of stigma. Initial psychometric analysis revealed that the total scale and subscales are internally consistent and reliable (.78 ≤ Cronbach’s α ≤ .94) [26].

The ILAS subscales were first translated into German by the first author. The ILAS subscales were then back-translated into English by a native English speaker fluent in German. Results were compared with the original version and discrepancies were discussed until consensus was reached. The original meaning of the items was conserved as much as possible in the German translation, only the instructions were slightly modified to highlight that the questions referred to study participants’ abortion after diagnosis of fetal anomaly. As the aim of this study was to evaluate the psychometric properties of the already established ILAS scale in a new population, piloting of the German version of the ILAS scale was not deemed necessary.

#### Depression and self-esteem

In order to investigate criterion validity of the ILAS, participants were given measures of depressive symptoms and self-esteem. We expected a positive relationship between the level of abortion stigma and depressive symptoms and a negative relationship between the level of abortion stigma and self-esteem, respectively. The German versions of the Revised Beck Depression Inventory (BDI-II) [39,40] and the Rosenberg Self-Esteem (RSE) scale [41–43] were used. Total scores were calculated for both measures, with higher scores reflecting higher levels of depressive symptoms and self-esteem. The German versions of the BDI-II and RSE scale have been shown to be reliable and valid. Internal consistency of both scales in the present data was excellent to good (RSE, Cronbach’s α = .88; BDI-II, Cronbach’s α = .92).

#### Secrecy

Participants also completed a measure of secrecy to further investigate the validity of the ILAS. We expected that higher levels of abortion stigma are associated with stronger intentions to keep the abortion a secret. For this purpose, we translated and adapted the Secrecy scale originally developed by Link et al. to assess secrecy intentions among people living with mental illness [44]. The scale used in this study consisted of all the 5 Likert-scale items of the original version (ranging from 1 –strongly disagree to 4—strongly agree), but was modified to measure the extent to which respondents’ would conceal their abortion experience in different social situations (for example: “If a woman has had a later abortion (after diagnosis of a fetal anomaly), the best thing to do is keep it a secret”). Internal consistency was acceptable (Cronbach’s α = .71). Total scores were calculated, with higher scores reflecting more secrecy intentions.

### Data analysis

All data analyses were performed using SPSS, version 23/24 and the significance level was set to α = .05.

#### Treatment of missing data

All participants who had answered every item of the ILAS scale were included in the analysis (n = 130). Listwise deletion (i.e. excluding the entire observation in the case of missing data on one or more measures) was used to account for missing data on the remaining measures to ensure comparability across results. Overall, the missing data rate was low (1.2%) and Little’s MCAR-test was non-significant (Chi-Square = 334.724, D*f* = 325, *p* = .34) indicating that the data were missing completely at random. Under this condition, bias that can result from listwise deletion is likely to be negligible [45,46].

#### Factor structure and internal consistency

We chose an exploratory factor analysis (EFA) over a confirmatory factor analysis (CFA) to investigate the latent structure of the ILAS scale for several methodological considerations. First, the sample used in this study differed from the original sample of Cockrill et al. [26] with regard to two important characteristics (socio-cultural setting and focus on abortion after diagnosis of fetal anomaly), which could be associated with a different underlying factor structure. Second, the relatively small sample size of this study and the relatively small number of two items that make up the community condemnation subscale can lead to unstable parameter estimates when conducting a CFA. A minimum sample size of 200 and a minimum number of four indicators/items per latent variable/subscale have been recommended [47]. Based on the analyses of Cockrill et al. [26], we conducted an exploratory factor analysis (EFA) with orthogonal rotation. The Principal Axis Factor method with Varimax rotation was used. Parallel analysis (using an SPSS macro [48]) and the eigenvalue > 1 criterion were used to determine the number of factors, in conjunction with a visual inspection of the scree plot [47]. Item loadings > .40 were considered substantive [49]. Cronbach’s α was calculated to investigate internal consistency for the ILAS subscales, with Cronbach’s α ≥ .70 deemed acceptable [49].

#### Test-retest reliability

The first 100 participants to return the questionnaire were sent the ILAS scale again three weeks after completion of the first questionnaire to estimate test-retest reliability of the ILAS scale. Three weeks was chosen as lower boundary of the test-retest interval to minimize carry-over effects induced by the first testing. The time for returning the second questionnaire was restricted to five weeks as the isolation subscale included some items that may be sensitive to change over time (e.g. “I have had a conversation with someone I am close with about my abortion“). Test-retest reliability was determined by calculating a two-way mixed-effects ICC with absolute measure of agreement (ICC (2,2) [50]. An ICC less than .40 was interpreted as poor, an ICC between .40 and .59 was interpreted as fair, an ICC between .60 and .74 was interpreted as good and an ICC greater than .75 was interpreted as excellent [51].

#### Item and scale characteristics

On the item level, we investigated the percentage of missing data as indicator of item acceptance and mean (*M*), standard deviation (*SD*) and percentage of answers in the lowest category as indicators of response distribution. Additionally, we examined whether the whole range of possible response categories was used. Corrected item-total-correlations were computed and expected to be greater than .40 [52]. *M*, *SD*, skewness, and kurtosis were calculated as indicators of distribution of responses on the ILAS subscales.

#### Stigma correlates

We calculated partial correlations using Spearman’s *ρ* to examine the relationship between the ILAS subscales and the measures of depression, self-esteem, and secrecy. Correlation coefficients of ≥ .10, ≥ .30 and ≥ .50 were interpreted as small, moderate and large effect sizes, respectively [53]. To account for potential effects of time, time since abortion was included as control variable.

#### Multivariate regression analyses

We explored factors associated with abortion stigma after diagnosis of fetal anomaly by conducting multivariate ordinary least squares (OLS) regression analyses. We chose general sociodemographic variables as well as variables that had been associated with the abortion experience and related stigma in previous research [11,26,54]. In a first step, bivariate associations of age at time of the abortion (years), religious affiliation (yes/no), education (low vs. all others), income (low vs. all others), reproductive history before the abortion (previous live births yes/no and abortions yes/no), gestational age (weeks), perceived partner support at the time of the abortion (1 –no support to 5—strong support) and perceived low survival chance of the fetus (yes/no) with the ILAS subscales were examined to identify significant correlations. In a second step, variables with significant associations were entered simultaneously into regression models with the ILAS subscales as dependent variables. The following variables were entered in the final regression models: Age at time of the abortion, income, gestational age, perceived low survival chance of the fetus and perceived partner support. Time since the abortion (years) was included as control variable in all regression models. Some of the regression models showed indication of heteroscedasticity and non-normal distributions of residuals. Therefore, we calculated and interpreted bootstrapped confidence intervals of regression coefficients to minimize bias in the inferential statistics using the bias-corrected and accelerated method [49,55,56]. Regression coefficients were considered different from zero when the 95% confidence interval (95% CI) did not include zero.

## Results

### Sample characteristics

The register search resulted in a total of 395 women who had had an abortion after diagnosis of fetal anomaly between September 2008 and January 2015 and who were eligible for study inclusion. Valid addresses were available from 318 women, who were contacted between October 2015 and February 2016. One-hundred and forty-eight (148) women gave informed consent and returned the questionnaire (response rate: 46.5%). Sociodemographic characteristics of participants at the time of study participation and reproductive health variables related to the abortion are presented in Table 1 and Table 2.

**Table 1:** Sociodemographic characteristics at the time of study participation.

		Total (N = 148)
		n (%)^a^
**Age, years**	≤ 29	20 (13.5)
	30–34	54 (36.5)
	35–39	49 (33.1)
	≥ 40	25 (16.9)
**Education**	≥ 12 years	90 (60.8)
	< 12 years	58 (39.2)
**Employment status**	Employed	102 (68.9)
	Parental leave	35 (23.6)
	Other	11 (7.4)
**Household income**	< 1500 EUR	38 (26.0)
	≥ 1500	108 (74.0)
**Religious affiliation**	None	109 (73.6)
	Protestant	32 (21.6)
	Other	7 (4.7)

Notes.

^a^ Percentages are calculated from valid case.

**Table 2:** Reproductive health variables related to the abortion.

		Total (N = 148)
		n (%)^a^
**Age at abortion**	≤24	9 (6.1)
	25–29	55 (37.2)
	30–34	46 (31.1)
	35–38	30 (20.3)
	≥40	8 (5.4)
**Gestational age, weeks**	15–26 (2nd trimester)	128 (86.5)
	>26 (3rd trimester)	20 (13.5)
**Type of fetal malformation**^b^	Trisomy 21	24 (16.2)
	Multiple syndrome	17 (11.5)
	Spina bifida	12 (8.1)
	Other	95 (64.2)
**Perceived low survival chance of fetus**^c^	Yes	54 (38.0)
	No	88 (62.0)
**Previous live birth**	Yes	65 (32.4)
	No	83 (60.7)
**Previous abortion**	Yes	13 (8.8)
	No	135 (91.2)
**Partnership at abortion**	Yes	146 (98.6)
	No	2 (1.4)
**Partner support**	Strong/very strong^d^	120 (82.8)
**Time since abortion, years**	*M* = 3.63 (*SD* = 1.88, range: 1.08–7.42)	

Notes.

^a^ Percentages are calculated from valid cases.

^b^ Only the first three most prevalent categories are presented.

^c^ Information obtained by coding open-ended responses on women’s reason for abortion after diagnosis of fetal anomaly.

^d^ Answer options ranged from 1 –no support at all to 5- very strong support (M = 4.17, SD = 1.07).

The majority of participants had no religious affiliation (73.6%) and were highly educated (60.8%). The mean age at the time of the abortion was 31.59 years (*SD* = 5.01 years) and on average 3.63 years (*SD* = 1.88 years) had passed since the event at the time of the assessment. Most women had had their abortion in the second trimester (86.5%) (gestational age, weeks: M = 22.22, SD = 4.45, range: 15 to 39) and the most frequently reported fetal anomaly was Trisomy 21 (16.2%). The majority of women were in a partnership at the time of the abortion (98.6%) and had not experienced an abortion before (91.2%).

### Factor structure and internal consistency

An EFA was conducted with the items of the German Version of the ILAS scale [26]. The results of the parallel analysis suggested the extraction of seven factors; however, only four factors with eigenvalues > 1 were identified. Buehner [47] argues that a principal axis factor analysis with parallel analysis (the approach used in this study) usually extracts a number of dispensable factors and suggest comparing the results to a principal component analysis (PCA) with parallel analysis. In the present sample, a parallel analysis based on a PCA indicated the extraction of four factors, which corresponded to the four factors with eigenvalue > 1 identified in the initial EFA. The factors were confirmed by visual inspection of the Scree-Plot and were thus retained. The allocation of items to factors suggested by the EFA was the same as in the original ILAS scale. Hence, the factors identified in this study replicated the worries about judgment subscale (19.0% variance explained), the isolation subscale (18.5% variance explained), the self-judgment subscale (16.6% variance explained) and the community condemnation subscale (8.6% variance explained) of the original ILAS scale. Together, the factors explained 62.7% of the total item variance. All primary factor loadings were substantive (.51 to .86) (see Table 3). One item of the worries about judgment subscale (item 6) cross-loaded relatively high (.39) on the self-judgment subscale. However, as the cross-loadings were still below the threshold of ≤ .40 and the primary factor loading of this item was relatively high (.62), the item was included in the primary factor only. Cronbach’s α for the subscales well exceeded the acceptable lower limit of .70 for all the subscales (see Table 4). The subscales were significantly inter-correlated, and the highest correlation was observed between the worries about judgment and the self-judgment subscale (*ρ =* .60) (see Table 5).

**Table 3:** Item characteristics.

						EFA			
	% missing	M	SD	% in lowest category	Item-total-correlation^f^	Factor I	Factor II	Factor III	Factor IV
**Worries about judgment**^a^									
Other people might find out about my abortion. (Item 1)	<1	0.68	.93	56.9	.62	**.56**	.23	.24	.10
My abortion would negatively affect my relationship with someone I love. (Item 2)	<1	0.47	.87	72.3	.70	**.53**	.14	.35	.36
I would disappoint someone I love.(Item 3)	<1	0.47	.86	71.5	.75	**.61**	.19	.32	.34
I would be humiliated. (Item 4)	1.4	0.62	.93	61.5	.73	**.79**	.11	.10	.17
People would gossip about me. (Item 5)	<1	0.90	1.00	44.6	.78	**.81**	.14	.30	.03
I would be rejected by someone I love. (Item 6)	<1	0.52	.86	66.9	.77	**.62**	.19	.39	.31
People would judge me negatively. (Item 7)	<1	0.88	1.02	46.2	.81	**.80**	.15	.24	.09
**Isolation**									
I have had a conversation with someone I am close with about my abortion. (Item 8)^b^^,^ ^e^	<1	0.52	.70	57.7	.67	.08	**.71**	.33	.06
I was open with someone that I am close with about my feelings about my abortion. (Item 9)^b^^,^ ^e^	<1	0.69	.80	47.7	.69	.03	**.75**	.01	.12
I felt the support of someone that I am close with at the time of my abortion. (Item 10)^b^^,^ ^e^	<1	0.52	.87	67.7	.70	.17	**.74**	.03	.17
I can talk to the people I am close with about my abortion. (Item 11)^c^^,^ ^e^	<1	0.78	1.03	53.8	.76	.21	**.78**	-.13	-.01
I can trust the people I am close to with information about my abortion. (Item 12)^c^^,^ ^e^	<1	0.69	.94	56.2	.75	.16	**.77**	.20	-.05
When I had my abortion I felt supported by the people I was close with. (Item 13)^c^^,^ ^e^	<1	0.80	1.06	52.3	.78	.15	**.79**	.24	.08
**Self-judgment**^**c**^									
I felt like a bad person. (Item 14)	0	2.08	1.39	17.7	.77	.32	.09	**.75**	.06
I felt confident I had made the right decision. (Item 15)^e^	0	1.23	1.14	31.5	.53	.17	.02	**.51**	.20
I felt ashamed about my abortion. (Item 16)	0	1.15	1.22	38.5	.75	.33	.08	**.73**	.18
I felt selfish. (Item 17)	0	1.52	1.36	28.5	.68	.17	.03	**.75**	.04
I felt guilty. (Item 18)	0	2.13	1.31	13.8	.76	.22	.06	**.77**	.10
**Community condemnation**^d^									
Abortion is always wrong. (Item 19)	10.8	0.59	.85	57.7	.70	.17	.12	.12	**.66**
Abortion is the same as murder. (Item 20)	10.8	0.48	.86	68.5	.70	.23	.08	.22	**.86**

Notes. % of missing based on N = 148, all other data based on n = 130

^a^ Answer options were “not worried,” “a little worried,” “quite worried” and “extremely worried”

^b^ Answer options were “never,” “once,” “more than once” and “many times”

^c^ Answer options were “strongly disagree,” “disagree,” “neither agree nor disagree,” “agree” and “strongly agree”

^d^ Answer options were “no one”, “a few people”, “about half the people”, “many people” or “most people”

^e^ Item was reverse coded

^f^ Corrected item-total-correlation on subscale level.

**Table 4:** Scale characteristics.

	M (SD)^a^	SD^a^	Range^a^	Skewness^a^	Kurtosis^a^	Cronbach’s α^a^	Test-retest^b^^,^^c^
**Worries about Judgment**	0.65	0.75	0–3	1.38	1.43	.91	.89
**Isolation**	0.67	0.74	0–3.5	1.49	2.50	.90	.57
**Self-judgment**	1.62	1.04	0–4	0.38	-0.79	.87	.92
**Community Condemnation**	0.54	0.79	0–4	1.72	2.75	.83	.68

Notes.

^a^ n = 130

^b^ n = 57

^c^ Test-retest interval: *M* = 28.6 days (*SD* = 3.6, range: 24–36).

**Table 5:** Stigma correlates.

	Worries about judgment	Isolation	Self-judgment	BDI-II^a^	RSE^a^	Secrecy^a^
**Worries about Judgment**	-	-	-	.51***	-.39***	.41***
**Isolation**	.39***	-	-	.19*	-.30***	.23**
**Self-judgment**	.60***	.19*	-	.56***	-.38***	.33***
**Community condemnation**	.37***	.20*	.32***	.33***	-.28***	.27**

Notes. n = 127

*** *p* < .001

** *p* < .01

*p < .05

^a^ Controlled for time since abortion.

### Test-retest reliability

Of the 100 participants who were contacted for the retest, 80 returned the questionnaire (response rate: 80.0%) The test-retest analyses were based on all participants who had completed the second ILAS scale three to five weeks after the first one (*n* = 57; test-retest interval in days: 24 to 36, *M* = 28.6, *SD* = 10.76). Test-retest stability was excellent for the worries about judgment subscale (ICC(2,2) = 0.89) and the self-judgment subscale (ICC(2,2) = 0.92), good for the community condemnation subscale (ICC(2,2) = 0.68) and fair for isolation subscale (ICC(2,2) = 0.57).

### Item and scale characteristics

The item and scale characteristics of the German Version of the ILAS scale are presented in Table 3 and Table 4, respectively. On the subscale level, all corrected item-total-correlations exceeded .40. However, several items (items 8, 9, 10, 19) were found to correlate below .40 with the total scale. Consequently, we decided not to compute a total score for the ILAS.

The rate of missing responses for the items of the subscales worries about judgment, isolation, and self-judgment was low (≤ 1.4%), indicating high item acceptance. On both items of the community condemnation subscale, 10.8% of responses were missing. A descriptive analysis of open-ended responses revealed that some participants found it difficult to evaluate abortion-related attitudes within a social unit as large as their city or town. Responses to the worries about judgment subscale (M = 0.65, SD = 0.75, range: 0–3), the isolation subscale (M = 0.67, SD = 0.74, range: 0–3.5), and the community condemnation subscale (M = 0.54, SD = 0.79, range: 0–4) were concentrated on the low scale end. Between 44.6% and 72.3% of participants chose the lowest response category of items on these subscales (see Table 3), indicating that substantial proportions of respondents did not experience abortion stigma as measured by items on these subscales. Responses to the items of the self-judgment subscale were roughly normally distributed and centered slightly below the middle of the scale (*M* = 1.62, *SD* = 1.04). Across all items, all response categories were used by the respondents.

### Stigma correlates

Information on the associations between the ILAS subscales and related constructs are presented in Table 5. The ILAS subscales were positively associated with depressive symptoms (.19 ≤ *ρ* ≤ .56, *ps* ≤ .02) and secrecy (.23 ≤ *ρ* ≤ .41, *ps* ≤ .001), and negatively associated with self-esteem (-.28 ≤ *ρ* ≤ -.39, *ps* ≤ .001). For the worries about judgment subscale and self-judgment subscale, the effect size of the correlation coefficients was medium to large. By contrast, the correlation coefficients of the isolation subscale and the community condemnation subscale were mostly small.

### Multivariate regression analyses

The results of the final regression models are displayed in Table 6. Women who perceived that their fetus had a low survival chance scored lower on the worries about judgment subscale (*β* = -.20, *p* = .024, 95% BcaCI: -0.54 –-0.08). Higher partner support was associated with lower scores on the isolation subscale (*β* = -.34, *p* = .005, 95% BcaCI: -0.38 –-0.10). Higher gestational age was associated with higher scores on the self-judgment subscale (*β* = .20, *p* = .016, 95% BcaCI: 0.01–0.09) whereas women who perceived that their fetus had a low survival chance scored lower on the self-judgment subscale (*β* = -.28, *p* = .009, 95% BcaCI: -0.95 –-0.27). Women with high partner support scored lower on the community condemnation subscale (*β* = -.28, *p* = .015, 95% BcaCI: -0.38 –-0.04).

**Table 6:** Coefficients (B, β) and bootstrapped confidence intervals of variables associated with abortion stigma.

	Worries about judgment		Isolation		Self-judgment		Community Condemnation	
Variable	B (95% CI)	β	B (95% CI)	β	B (95% CI)	β	B (95% CI)	β
Age	-0.02 (-0.05–0.004)	-.13	-0.02 (-0.04 – -0.007)	-.11	-0.02 (-0.07–0.02)	-.10	0.007 (-0.03–0.04)	.04
Income, *< 1500 Euro*	0.14 (-0.18 – 0.47)	.09	0.27 (-0.05 – 0.58)	.16	0.36 (-0.10–0.84)	.15	0.35 (-0.01–0.73)	.19
Gestational age	0.02 (-0.009 – 0.04)	.10	-0.03 (-0.06 – 0.008)	-.16	0.05 (0.01–0.09)	.20*	0.02 (-0.01–0.05)	.11
Perceived low survival chance, *yes*	-0.30 (-0.54 – -0.08)	-.20*	-0.03 (-0.28 – 0.25)	-.02	-0.59 (-0.95 –-0.27)	-.28*	0.01 (-0.27–0.27)	.01
Partner support	-0.11(-0.24 – 0.001)	-.16	-0.23 (-0.38 –-0.10)	-.34*	-0.07 (-0.26–0.11)	-.07	-0.21 (-0.38 –-0.04)	-.28*
Time since abortion	-0.06 (-0.12– -0.001)	-.14	0.03 (-0.05–0.11)	.08	-0.06 (-0.16–0.05)	-.10	-0.04 (-0.12–0.04)	-.09
**R**^**2**^	.13		.23		.19		.16	

Notes. n = 121; B regression coefficient, β standardized regression coefficient, CI confidence interval, 95% CI based on 1000 bootstrap samples

*95% CI did not include zero.

## Discussion

Stigma can interfere with women’s psychological adjustment to an abortion. However, there is a lack of valid measures to assess stigma in the specific case of abortion after diagnosis of fetal anomaly. The first goal of this study was to validate a measure of abortion stigma (the ILAS scale) in a sample of women who have had an abortion after diagnosis of fetal anomaly in a German setting. Results suggest that the ILAS scale measures stigma across four dimensions or subscales in this population, comparable to the original version of the scale. All subscales showed satisfactory reliability and validity, except for the isolation subscale which had limited test-retest reliability.

An EFA produced an acceptable single structure solution, indicating that the items were related to four latent dimensions which corresponded to the subscales of the original ILAS scale: the worries about judgment subscale, the isolation subscale, the self-judgment subscale and the community condemnation subscale. The results of the present study did not support the calculation of a total score for the German version of the ILAS scale. While Cronbach’s α showed that the subscales are internally consistent, test-retest reliability was only fair for the isolation subscale. The exact reason for this remains unknown, but it is possible that the first assessment of isolation triggered introspective thoughts about the degree of isolation experienced. Such thoughts could have led to a decrease in feelings of isolation by the time of the re-test, or caused women to seek support before the re-test, thus changing their experience of isolation. Hence, the isolation subscale of the German Version of the ILAS scale should be used with caution in women who have had an abortion after diagnosis of fetal anomaly. Scores on the subscales were positively skewed and concentrated on the low scale ends or around the middle of the scale, indicating that substantial proportions of participants reported low levels of stigma. However, subscale scores varied and all response categories were used by respondents supporting the conclusion that the subscales are appropriate to capture women’s experience of abortion stigma.

Compared to the scores reported by women having abortions for various reasons in the original ILAS scale development study [26], women in this sample reported lower levels of stigma across all subscales. This observation supports the assumption that women who have abortions after diagnosis of fetal anomaly experience less stigma than for example women having abortions for socio-economic reasons, which might be in part a result of stronger public support for abortion after diagnosis of fetal anomaly [32–34]. However, the discrepant stigma scores observed in this sample and in the sample interviewed by Cockrill et al. [26] may also be related to the diverging construction and reproduction of abortion stigma in a U.S. and a German setting. In contrast to the ongoing controversial discussion of abortion within the U.S. public discourse [19], the abortion debate in Germany has largely subsided after a climax of abortion-rights mobilization in the mid-1990s [57]. These circumstances may generally mitigate stigma experienced by women having abortions in a German setting.

As in the development study by Cockrill et al. [26], our analyses suggest that the ILAS subscales assess inter-related dimensions of abortion stigma. The strongest relationship was found between worries about judgment subscale and the self-judgment subscale. The interdependence of these dimensions is theoretically meaningful, as the conceptual framework of abortion stigma explicitly states that internalized stigma (as measured by the self-judgment subscale) is a result of perceived stigma (as measured by the worries about judgment subscale) [22,23]. Vice versa, negative self-judgment that may have arisen from women’s feelings of responsibility for their fetus as well as personal moral conflicts related to the abortion [7,19] could have influenced their perception of negative judgment from others.

The items of the community condemnation subscale showed a relatively high rate of missing responses, indicating that they were not as well accepted by respondents in the present sample. Several participants stated that they found it difficult to evaluate the incidence of negative abortion-related attitudes in their communities. As noted above, this might be related to a lack of public discussion of abortion in Germany, leaving participants with little information to evaluate abortion-related attitudes at the community level. Relatedly, the rather broad definition of community implied by the subscale’s instruction (“How much of your community (city or town) held the following beliefs?”) might have limited participants’ access to information. Defining community as a smaller social unit such as women’s familiar social circles (e.g., family, friends, or colleagues) might increase the likelihood of valid responses when administering the subscale in future studies.

As hypothesized on the basis of theory and previous studies [24–27,29], we found that higher levels of abortion stigma as measured by the subscales of the German version of the ILAS scale were significantly associated with more depressive symptoms, lower self-esteem, and more secrecy intentions. However, the pattern of associations between the ILAS subscales and related constructs was not consistent with regard to the strength of associations. We observed medium to large effects for the worries about judgment subscale as well as the self-judgment subscale, but mostly small effects for the isolation subscale and the community condemnation subscale. This finding could reflect variability regarding the relevance of the different dimensions of abortion stigma for women’s psychological experience in the context of an abortion after diagnosis of fetal anomaly. The community condemnation subscale provides a rather objective measurement of perceptions of stigma without referring to an immediate threat to the self, which might have reduced associations with depression, self-esteem, and secrecy. With regard to the isolation subscale, the current set of items did not differentiate between women’s experiences of support from their partner and other sources. Hence, the items might have failed to capture variability in women’s experiences of isolation if respondents related them primarily to perceived support from their partner. The vast majority of women in this study were in a partnership at the time of their abortion and reported high partner support. However, study results indicate that the quality of support received from other sources such as friends or relatives can vary greatly [58]. Hence, modifying the subscale to include items that clearly differentiate between support sources might lead to a more nuanced assessment of isolation experienced by women having abortions after diagnosis of fetal anomaly.

Future studies adapting the ILAS scale for women having abortions after diagnosis of fetal anomaly should generally consider adding items/subscales that assess features of reproductive stigma specifically relevant to this population. For example, stigma might stem from notions of reproductive failure associated with the diagnosis of fetal anomaly, which may cause women to blame themselves for the anomaly [7]. Assessment of women’s experiences of stigma in interaction with healthcare providers may also be informative, as women have reported worries about judgment as a significant concern when receiving abortion care after diagnosis of fetal anomaly [59].

This study further sought to explore sociodemographic and abortion-related factors associated with women’s experiences of stigma in the context of abortion after diagnosis of fetal anomaly. A number of situational factors related to the abortion were associated with women’s experiences of stigma, namely gestational age, perceived low survival chance of the fetus and perceived partner support. As there are no fixed cut-off values for the ILAS subscales [26], these associations provide preliminary information on groups at risk for stigma. Women whose decision to terminate the pregnancy was not informed by low survival chance of the fetus and women at a higher gestational age were at higher risk of perceiving stigma and/or experiencing self-judgment. It is possible that the element of choice which is considered central to an abortion might be more relevant when the fetus is perceived to be viable, increasing perceived and internalized abortion-related stigma. On the other hand, the status of personhood might be more easily attributed to a fetus at higher gestational age, which might lead affected women to view their abortion as less morally justified. Evidence for a protective effect of partner support on the individual level of abortion stigma was not unambiguous. While partner support was related to less isolation and less perceived stigma (as measured by the community condemnation subscale), it did not appear to buffer feelings of self-judgment among the women in our sample. Research indicates that women consider the decision for an abortion after diagnosis of fetal anomaly their own, even when consulting with their partner [7]. Subsequently, it might be possible that the stigma attached to the decision is perceived as an individual burden and not alleviated by partner support. However, further investigation on this issue is needed which should also consider whether partners are prepared to give support that specifically addresses stigma. Contrary to findings of other studies on abortion stigma [20], religious affiliation was not related to any dimension of abortion stigma in the present sample. The majority of respondents with a religious affiliation associated with the Protestant church in our sample (32/39). The Protestant church in Germany holds a rather liberal position towards abortion acknowledging women’s individual decision [60], which might in part explain the discrepant results of this study. It is also possible that the binary measure of religious affiliation was not sensitive enough to capture variation in women’s religiosity, which has been found to be an important factor influencing abortion-related attitudes [20,31].

### Limitations

This study has some limitations. Responses might have been influenced by recall bias, as participants were asked to recall details on their abortion after a mean time of 3.63 years. To account for potential effects of time, we entered time since abortion as control variable in all respective analyses. Time since abortion was not associated with scores on any subscale of the ILAS scale and the test-retest coefficients for the majority of subscales indicated that responses were reliably measured over a period of 3 to 5 weeks. Causal interpretation of the associations between stigma and other study variables is not possible due to our cross-sectional sample design. The measure of perceived low survival chance of the fetus was derived from coding a qualitative open-ended question on women’s reason for the abortion, limiting the validity of this measure. Women may have thought their fetus had a low survival chance but happened not to mention it in the original qualitative open-ended question. Lastly, our sample was drawn from one large clinic in Germany and had a response rate of 46.5%, limiting the generalizability of study results. While the response rate was comparable to a similar study among German women 2 to 7 years after abortion following diagnosis of fetal anomaly (49%, [9]), it might have introduced a selection bias. Participants experiencing high levels of stigma may have refrained from study participation to avoid confrontation with their emotions and negative judgment from others, which might have led to an underestimation of the degree of individual-level abortion stigma found in this study.

### Conclusions

The results of this study provide important information on the assessment of abortion stigma among women who have had an abortion after diagnosis of fetal anomaly. Although the findings need to be interpreted within the specific German context, they support the conclusion that the ILAS subscales worries about judgment, self-judgment and community condemnation are reliable and valid measures to explore stigma in this population. Use of the isolation subscale cannot be recommended without restrictions due its limited reliability and researchers should evaluate its application in light of their specific research questions. Adding items that differentiate between sources of stigma and that measure stigma associated with reproductive failure might lead to a more nuanced assessment of individual-level stigma among women having abortions after diagnosis of fetal anomaly. Further investigations of psychometric properties of the ILAS scale should also involve a sufficient sample size to facilitate the verification of the underlying factor structure using a CFA approach. Building on suggestions by Cockrill et al. [26], the ILAS subscales can be used to assess the influence of stigma on the psychological adjustment to an abortion after diagnosis of fetal anomaly, to further investigate contextual and individual characteristics that cause variability in the stigma experienced by affected women and to evaluate the outcome of programs that seek to decrease stigma.

## Supporting information

S1 File German translation of the ILAS scale. (DOCX)
