# Supplementary material for: Abortion after diagnosis of fetal anomaly: Psychometric properties of a German version of the individual level abortion stigma scale
Source: PLoS One. 2018 Jun 12;13(6):e0197986. doi: 10.1371/journal.pone.0197986 (PMC5997354; doi:10.1371/journal.pone.0197986)
Supplement: S1 File — (DOCX) [file pone.0197986.s001.docx]

| **Sorgen über Bewertungen**  Bei den folgenden Fragen geht es um die Dinge, über welche Sie sich zum Zeitpunkt Ihres Schwangerschaftsabbruchs Sorgen gemacht haben. Wählen Sie die Antwort aus, die am besten beschreibt, wie besorgt Sie gewesen sind. | | | | | |
| --- | --- | --- | --- | --- | --- |
|  | | **Nicht besorgt** | **Ein wenig besorgt** | **Ziemlich besorgt** | **Extrem besorgt** |
|  | Andere Menschen könnten von meinem Schwangerschaftsabbruch erfahren. |  |  |  |  |
|  | Mein Schwangerschaftsabbruch könnte die Beziehung mit einem mir sehr nahestehenden Menschen negativ beeinflussen. |  |  |  |  |
|  | Ich könnte jemanden enttäuschen, der mir sehr nahe steht. |  |  |  |  |
|  | Ich könnte gedemütigt werden. |  |  |  |  |
|  | Andere Menschen könnten hinter meinem Rücken über mich reden. |  |  |  |  |
|  | Jemand, der mir sehr nahe steht, könnte mich zurückweisen. |  |  |  |  |
|  | Andere Menschen könnten negativ über mich urteilen. |  |  |  |  |
|  |  | 0 | 1 | 2 | 3 |

| **Isolation**  Bei den folgenden Fragen geht es um das Reden über Ihren Schwangerschaftsabbruch mit engen Freunden und anderen Menschen, die Ihnen nahe stehen. Denken Sie dabei an Ihren letzten Schwangerschaftsabbruch. Wählen Sie die Antwort aus, welche Ihre Erfahrung am besten beschreibt. | | | | | |
| --- | --- | --- | --- | --- | --- |
|  | | **Nie** | **Einmal** | **Mehr als einmal** | **Oft** |
|  | Ich habe mich mit jemanden, der mir nahe steht, über meinen Schwangerschaftsabbruch unterhalten. |  |  |  |  |
|  | Ich habe jemanden, der mir nahe steht, meine Gefühle über meinen Schwangerschaftsabbruch offen gezeigt. |  |  |  |  |
|  | Ich hatte zum Zeitpunkt meines Schwangerschaftsabbruchs das Gefühl, von jemandem unterstützt zu werden, der mir nahe steht. |  |  |  |  |
|  |  | 0 | 1 | 2 | 3 |

|  | | **Trifft über-haupt nicht zu** | **Trifft nicht zu** | **Trifft teilweise zu** | **Trifft zu** | **Trifft voll-ständig zu** |
| --- | --- | --- | --- | --- | --- | --- |
|  | Ich kann mit den Menschen, die mir nahe stehen, über meinen Schwangerschaftsabbruch reden. |  |  |  |  |  |
|  | Ich kann den Menschen, die mir nahe stehen, Informationen über meinen Schwangerschaftsabbruch anvertrauen. |  |  |  |  |  |
|  | Ich habe mich zum Zeitpunkt meines Schwangerschaftsabbruchs von den Menschen, die mir nahe standen, unterstützt gefühlt. |  |  |  |  |  |
|  |  | 0 | 1 | 2 | 3 | 4 |

| **Selbstbewertung**  Bei den folgenden Fragen geht es darum, wie Sie sich zum Zeitpunkt Ihres Schwangerschaftsabbruchs gefühlt haben. Bitte wählen Sie die Antwort aus, welche Ihre Gefühle am besten beschreibt. | | | | | | |
| --- | --- | --- | --- | --- | --- | --- |
|  |  | **Trifft über-haupt nicht zu** | **Trifft nicht zu** | **Trifft teilweise zu** | **Trifft zu** | **Trifft voll-ständig zu** |
|  | Ich habe mich wie ein schlechter Mensch gefühlt. |  |  |  |  |  |
|  | Ich war überzeugt, dass ich die richtige Entscheidung getroffen habe. |  |  |  |  |  |
|  | Ich habe mich für meinen Schwangerschaftsabbruch geschämt. |  |  |  |  |  |
|  | Ich habe mich egoistisch gefühlt. |  |  |  |  |  |
|  | Ich habe mich schuldig gefühlt. |  |  |  |  |  |
|  |  | 0 | 1 | 2 | 3 | 4 |

| **Verurteilung durch die Gemeinde**  Bei den folgenden Fragen geht es um die Gemeinde, in der Sie zum Zeitpunkt Ihres Schwangerschaftsabbruchs gelebt haben. Wie viele Menschen in Ihrer Gemeinde (Stadt oder Dorf) haben die folgenden Meinungen vertreten? | | | | | | |
| --- | --- | --- | --- | --- | --- | --- |
|  | | **Niemand** | **Ein paar Menschen** | **Ungefähr die Hälfte der Menschen** | **Viele Menschen** | **Die meisten Menschen** |
|  | Ein Schwangerschaftsabbruch ist immer falsch. |  |  |  |  |  |
|  | Ein Schwangerschaftsabbruch ist das gleiche wie ein Mord. |  |  |  |  |  |
|  |  | 0 | 1 | 2 | 3 | 4 |
